# Supplementary material for: Hairiness: the missing link between pollinators and pollination
Source: PeerJ. 2016 Dec 21;4:e2779. doi: 10.7717/peerj.2779 (PMC5180583; doi:10.7717/peerj.2779)
Supplement: Supplemental Information 4 [file peerj-04-2779-s004.pdf]

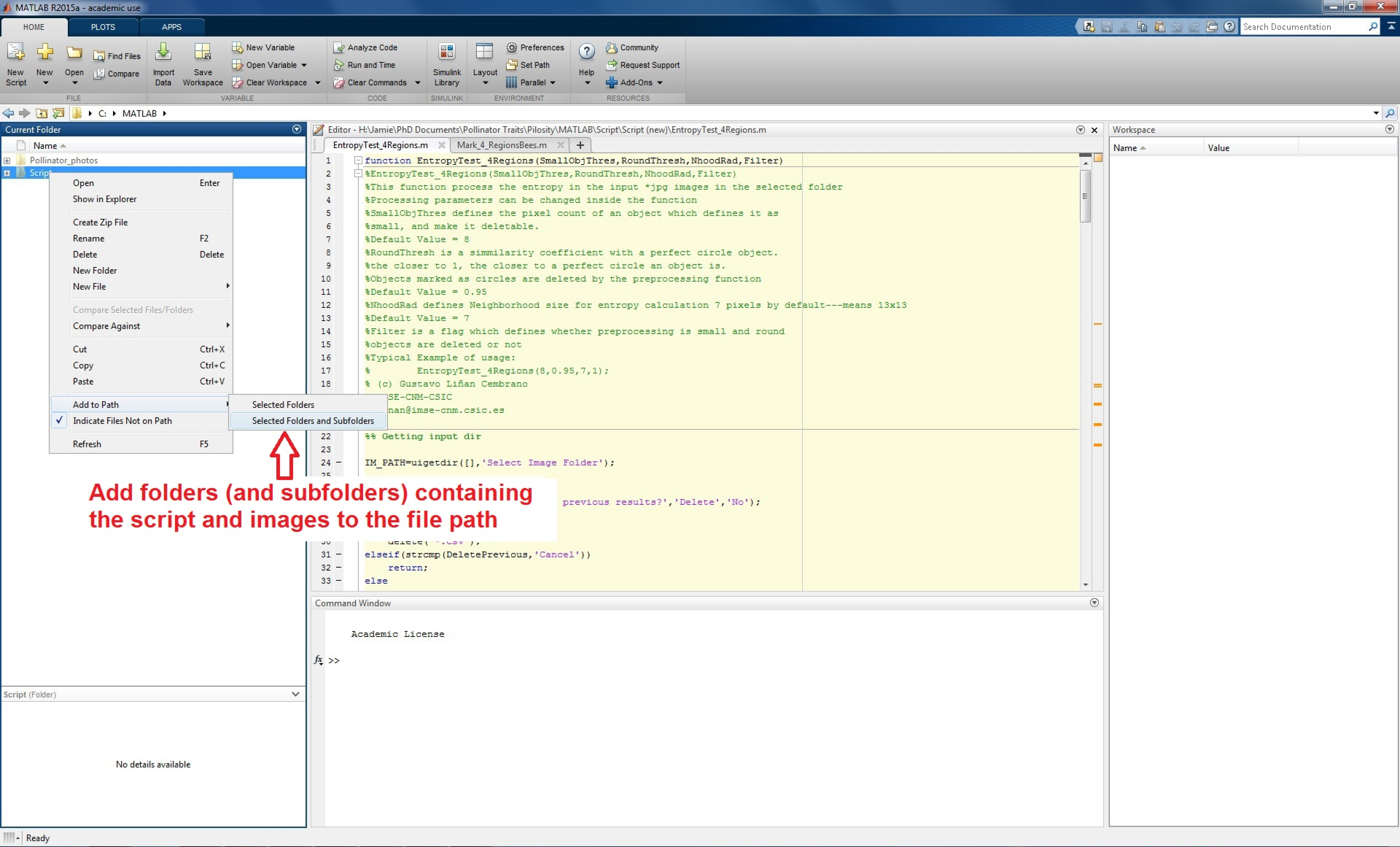

MATLAB R2015a - academic use
HOME
PLOTS
APPS
Search Documentation
New Script
New
Open
Find Files
Compare
FILE
Import Data
Save Workspace
New Variable
Open Variable
Clear Workspace
VARIABLE
Analyze Code
Run and Time
Clear Commands
CODE
Simulink Library
SIMULINK
Layout
Preferences
Set Path
Parallel
ENVIRONMENT
Help
Community
Request Support
Add-Ons
RESOURCES
C: ▸ MATLAB ▸
Current Folder
Name
Pollinator_photos
Script
Open Enter
Show in Explorer
Create Zip File
Rename F2
Delete Delete
New Folder
New File
Compare Selected Files/Folders
Compare Against
Cut Ctrl+X
Copy Ctrl+C
Paste Ctrl+V
Add to Path
Indicate Files Not on Path
Refresh F5
Selected Folders
Selected Folders and Subfolders
Editor - H:\Jamie\PhD Documents\Pollinator Traits\Pilosity\MATLAB\Script\Script (new)\EntropyTest_4Regions.m
EntropyTest_4Regions.m
Mark_4_RegionsBees.m
function EntropyTest_4Regions(SmallObjThres,RoundThresh,NhoodRad,Filter)
%EntropyTest_4Regions(SmallObjThres,RoundThresh,NhoodRad,Filter)
%This function process the entropy in the input *jpg images in the selected folder
%Processing parameters can be changed inside the function
%SmallObjThres defines the pixel count of an object which defines it as
%small, and make it deletable.
%Default Value = 8
%RoundThresh is a similarity coefficient with a perfect circle object.
%the closer to 1, the closer to a perfect circle an object is.
%Objects marked as circles are deleted by the preprocessing function
%Default Value = 0.95
%NhoodRad defines Neighborhood size for entropy calculation 7 pixels by default---means 13x13
%Default Value = 7
%Filter is a flag which defines whether preprocessing is small and round
%objects are deleted or not
%Typical Example of usage:
% EntropyTest_4Regions(8,0.95,7,1);
% (c) Gustavo Liñan Cembrano
%% Getting input dir
IM_PATH=uigetdir([],'Select Image Folder');
previous results?','Delete','No');
elseif(strcmp(DeletePrevious,'Cancel'))
return;
else
Command Window
Academic License
>>
Workspace
Name
Value
Script (Folder)
No details available
Ready
Add folders (and subfolders) containing the script and images to the file path

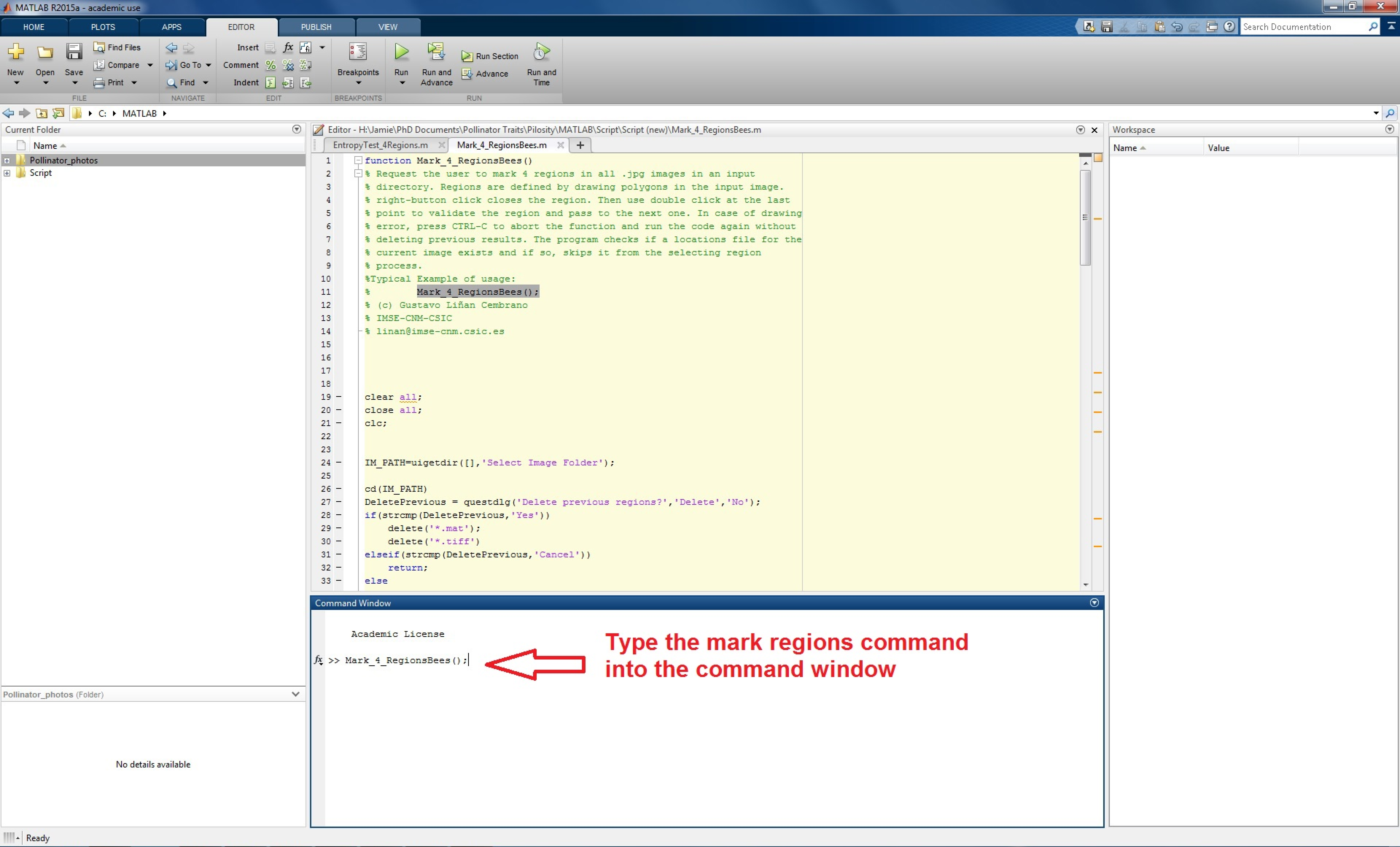
MATLAB R2015a - academic use
HOME
PLOTS
APPS
EDITOR
PUBLISH
VIEW
Search Documentation
New
Open
Save
Find Files
Compare
Print
FILE
Go To
Find
NAVIGATE
Insert
Comment
Indent
EDIT
Breakpoints
BREAKPOINTS
Run
Run and Advance
Run Section
Advance
Run and Time
RUN
C: ▸ MATLAB ▸
Current Folder
Name
Pollinator_photos
Script
Editor - H:\Jamie\PhD Documents\Pollinator Traits\Pilosity\MATLAB\Script\Script (new)\Mark_4_RegionsBees.m
EntropyTest_4Regions.m
Mark_4_RegionsBees.m
function Mark_4_RegionsBees()
% Request the user to mark 4 regions in all .jpg images in an input
% directory. Regions are defined by drawing polygons in the input image.
% right-button click closes the region. Then use double click at the last
% point to validate the region and pass to the next one. In case of drawing
% error, press CTRL-C to abort the function and run the code again without
% deleting previous results. The program checks if a locations file for the
% current image exists and if so, skips it from the selecting region
% process.
%Typical Example of usage:
% Mark_4_RegionsBees();
% (c) Gustavo Liñan Cembrano
% IMSE-CNM-CSIC
% linan@imse-cnm.csic.es
clear all;
close all;
clc;
IM_PATH=uigetdir([],'Select Image Folder');
cd(IM_PATH)
DeletePrevious = questdlg('Delete previous regions?','Delete','No');
if(strcmp(DeletePrevious,'Yes'))
delete('*.mat');
delete('*.tiff')
elseif(strcmp(DeletePrevious,'Cancel'))
return;
else
Workspace
Name
Value
Command Window
Academic License
>> Mark_4_RegionsBees();
Type the mark regions command into the command window
Pollinator_photos (Folder)
No details available
Ready

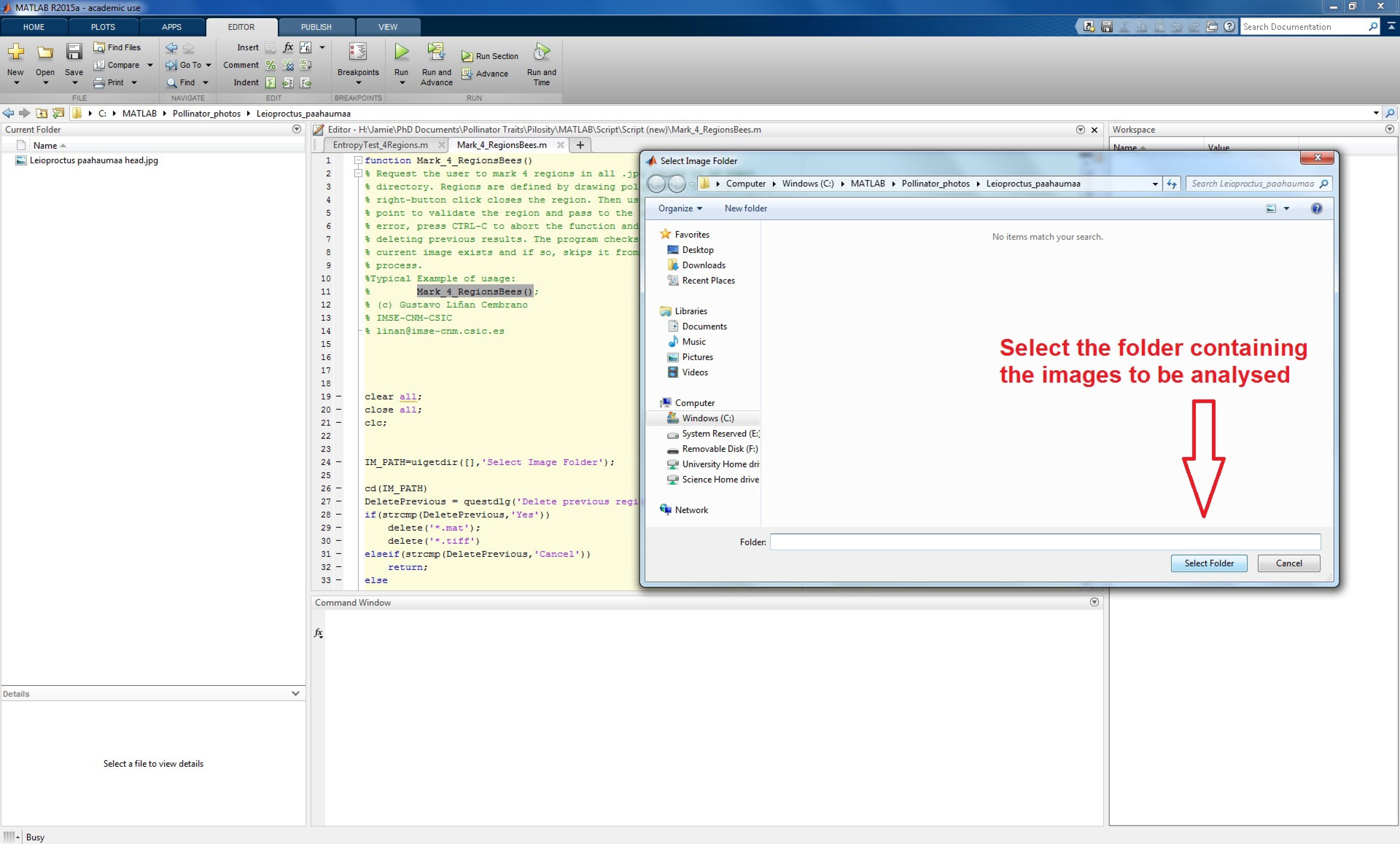
MATLAB R2015a - academic use
HOME
PLOTS
APPS
EDITOR
PUBLISH
VIEW
Search Documentation
C: ▸ MATLAB ▸ Pollinator_photos ▸ Leioproctus_paahaumaa
Current Folder
Leioproctus paahaumaa head.jpg
Editor - H:\Jamie\PhD Documents\Pollinator Traits\Pilosity\MATLAB\Script\Script (new)\Mark_4_RegionsBees.m
EntropyTest_4Regions.m
Mark_4_RegionsBees.m
function Mark_4_RegionsBees()
% Request the user to mark 4 regions in all .jp
% directory. Regions are defined by drawing pol
% right-button click closes the region. Then us
% point to validate the region and pass to the
% error, press CTRL-C to abort the function and
% deleting previous results. The program checks
% current image exists and if so, skips it from
% process.
%Typical Example of usage:
% Mark_4_RegionsBees();
% (c) Gustavo Liñan Cembrano
% IMSE-CNM-CSIC
% linan@imse-cnm.csic.es
clear all;
close all;
clc;
IM_PATH=uigetdir([],'Select Image Folder');
cd(IM_PATH)
DeletePrevious = questdlg('Delete previous regi
if(strcmp(DeletePrevious,'Yes'))
delete('*.mat');
delete('*.tiff')
elseif(strcmp(DeletePrevious,'Cancel'))
return;
else
Select Image Folder
Computer ▸ Windows (C:) ▸ MATLAB ▸ Pollinator_photos ▸ Leioproctus_paahaumaa
Organize
New folder
No items match your search.
Favorites
Desktop
Downloads
Recent Places
Libraries
Documents
Music
Pictures
Videos
Computer
Windows (C:)
Network
Folder:
Select Folder
Cancel
Select the folder containing the images to be analysed
Command Window
Workspace
Details
Select a file to view details
Busy

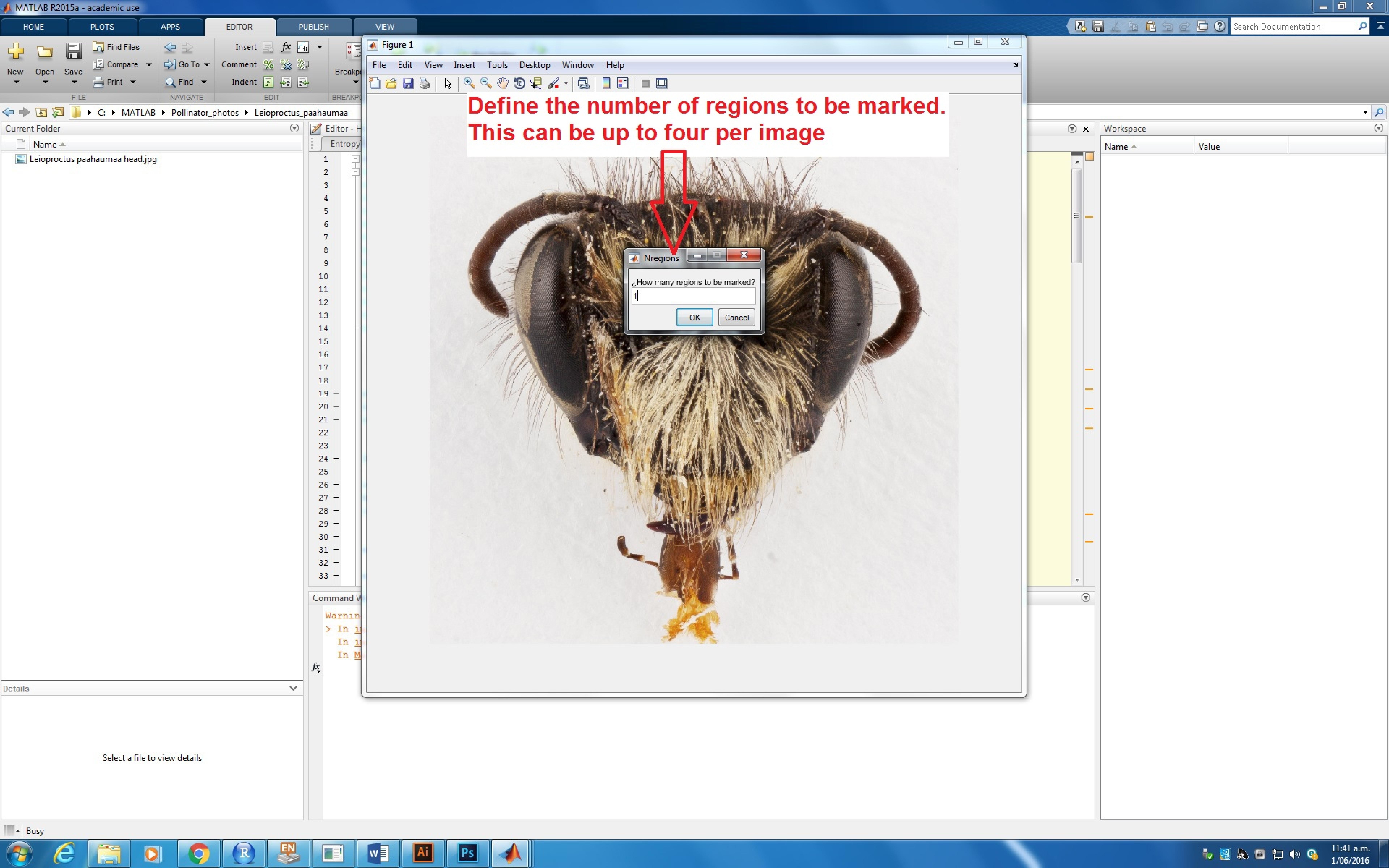
MATLAB R2015a - academic use
HOME
PLOTS
APPS
EDITOR
PUBLISH
VIEW
Search Documentation
Figure 1
File Edit View Insert Tools Desktop Window Help
Define the number of regions to be marked.
This can be up to four per image
Nregions
¿How many regions to be marked?
1
OK
Cancel
C: ▸ MATLAB ▸ Pollinator_photos ▸ Leioproctus_paahaumaa
Current Folder
Name
Leioproctus paahaumaa head.jpg
Details
Select a file to view details
Workspace
Name
Value
Command Window
Busy
11:41 a.m.
1/06/2016

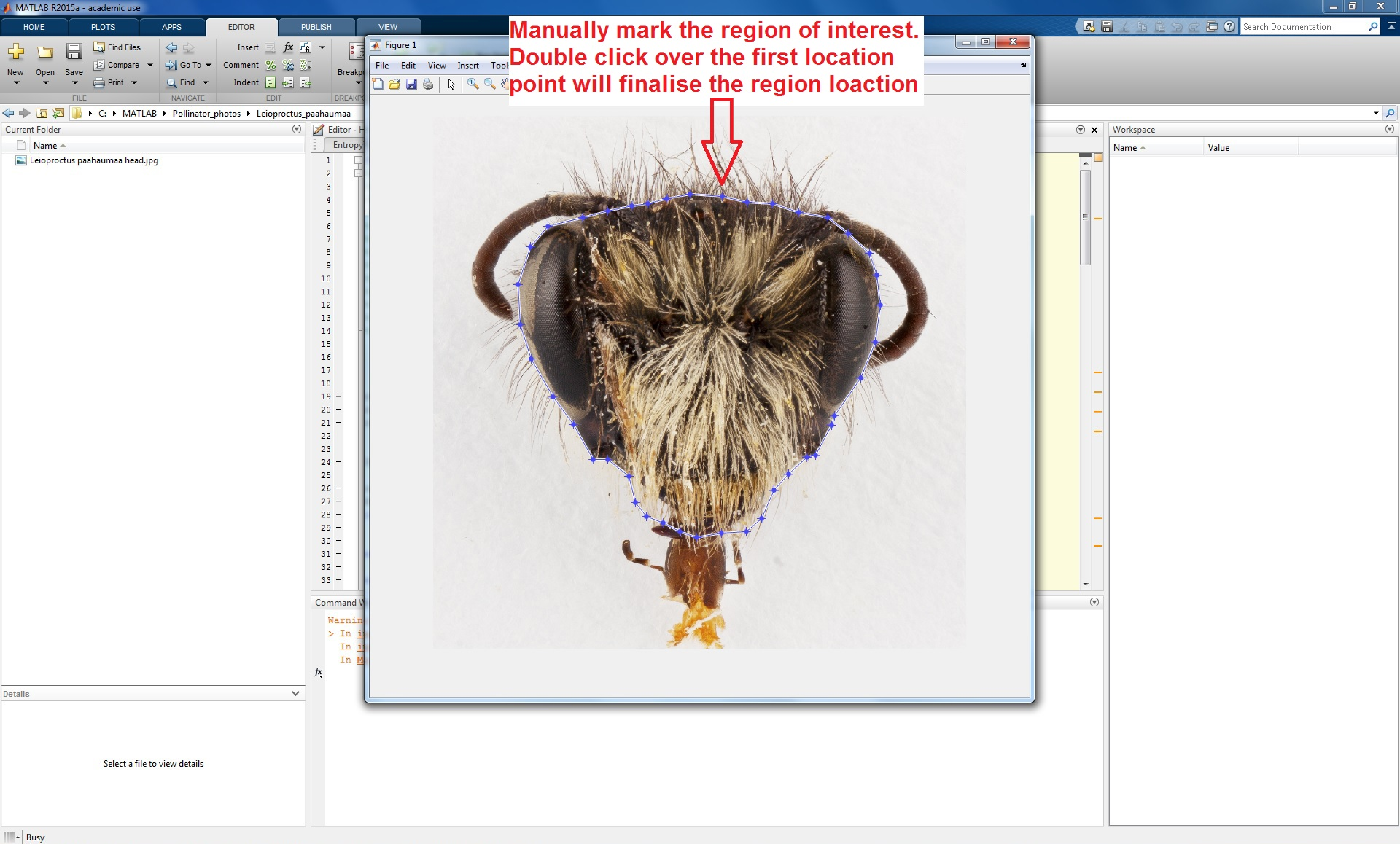
MATLAB R2015a - academic use
HOME
PLOTS
APPS
EDITOR
PUBLISH
VIEW
Manually mark the region of interest.
Double click over the first location
point will finalise the region loaction
Figure 1
File
Edit
View
Insert
Tool
New
Open
Save
Find Files
Compare
Print
Go To
Find
Insert
Comment
Indent
FILE
NAVIGATE
EDIT
C: ▸ MATLAB ▸ Pollinator_photos ▸ Leioproctus_paahaumaa
Current Folder
Name
Leioproctus paahaumaa head.jpg
Details
Select a file to view details
Workspace
Name
Value
Search Documentation
Busy

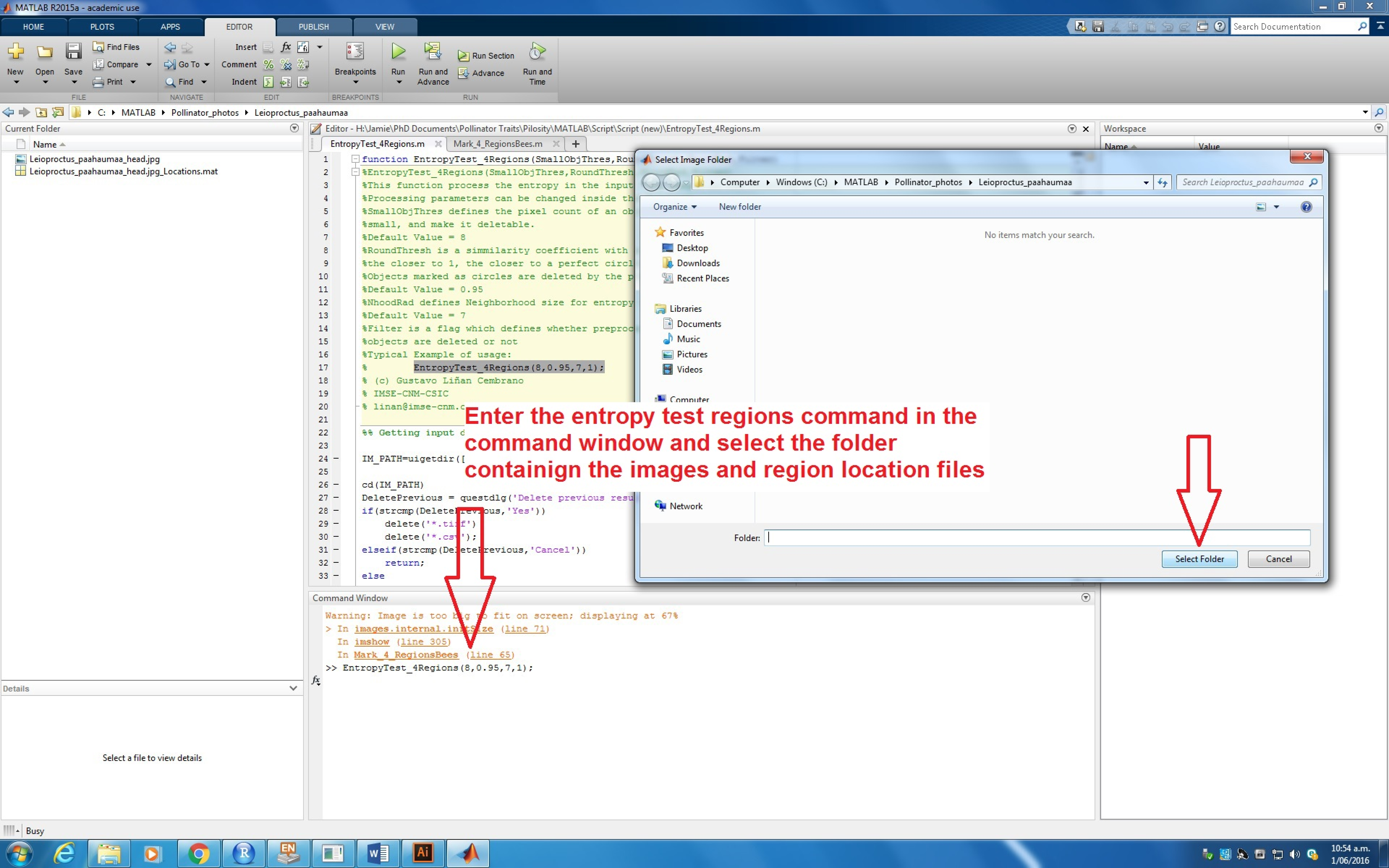
MATLAB R2015a - academic use
Select Image Folder
Computer ▸ Windows (C:) ▸ MATLAB ▸ Pollinator_photos ▸ Leioproctus_paahaumaa
No items match your search.
Folder:
Select Folder
Cancel
Enter the entropy test regions command in the command window and select the folder containign the images and region location files
Command Window
Warning: Image is too big to fit on screen; displaying at 67%
> In images.internal.initSize (line 71)
In imshow (line 305)
In Mark_4_RegionsBees (line 65)
>> EntropyTest_4Regions(8,0.95,7,1);

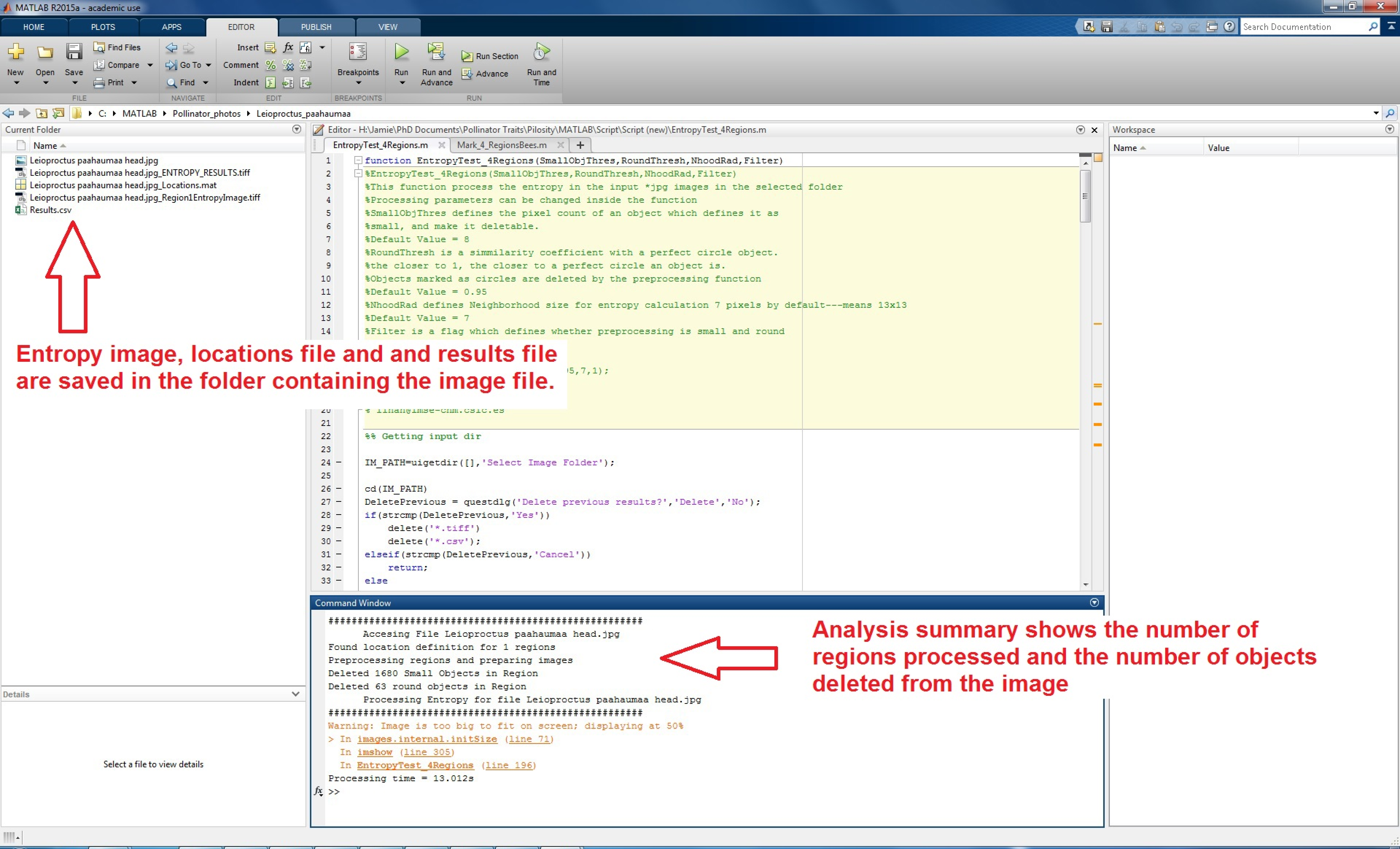
MATLAB R2015a - academic use
HOME
PLOTS
APPS
EDITOR
PUBLISH
VIEW
Search Documentation
New
Open
Save
Find Files
Compare
Print
FILE
Go To
Find
NAVIGATE
Insert
Comment
Indent
EDIT
Breakpoints
BREAKPOINTS
Run
Run and Advance
Run Section
Advance
Run and Time
RUN
C: ▸ MATLAB ▸ Pollinator_photos ▸ Leioproctus_paahaumaa
Current Folder
Name
Leioproctus paahaumaa head.jpg
Leioproctus paahaumaa head.jpg_ENTROPY_RESULTS.tiff
Leioproctus paahaumaa head.jpg_Locations.mat
Leioproctus paahaumaa head.jpg_Region1EntropyImage.tiff
Results.csv
Entropy image, locations file and and results file are saved in the folder containing the image file.
Details
Select a file to view details
Editor - H:\Jamie\PhD Documents\Pollinator Traits\Pilosity\MATLAB\Script\Script (new)\EntropyTest_4Regions.m
EntropyTest_4Regions.m
Mark_4_RegionsBees.m
function EntropyTest_4Regions(SmallObjThres,RoundThresh,NhoodRad,Filter)
%EntropyTest_4Regions(SmallObjThres,RoundThresh,NhoodRad,Filter)
%This function process the entropy in the input *jpg images in the selected folder
%Processing parameters can be changed inside the function
%SmallObjThres defines the pixel count of an object which defines it as
%small, and make it deletable.
%Default Value = 8
%RoundThresh is a similarity coefficient with a perfect circle object.
%the closer to 1, the closer to a perfect circle an object is.
%Objects marked as circles are deleted by the preprocessing function
%Default Value = 0.95
%NhoodRad defines Neighborhood size for entropy calculation 7 pixels by default---means 13x13
%Default Value = 7
%Filter is a flag which defines whether preprocessing is small and round
%% Getting input dir
IM_PATH=uigetdir([],'Select Image Folder');
cd(IM_PATH)
DeletePrevious = questdlg('Delete previous results?','Delete','No');
if(strcmp(DeletePrevious,'Yes'))
delete('*.tiff')
delete('*.csv');
elseif(strcmp(DeletePrevious,'Cancel'))
return;
else
Workspace
Name
Value
Command Window
##
Accesing File Leioproctus paahaumaa head.jpg
Found location definition for 1 regions
Preprocessing regions and preparing images
Deleted 1680 Small Objects in Region
Deleted 63 round objects in Region
Processing Entropy for file Leioproctus paahaumaa head.jpg
##
Warning: Image is too big to fit on screen; displaying at 50%
> In images.internal.initSize (line 71)
In imshow (line 305)
In EntropyTest_4Regions (line 196)
Processing time = 13.012s
>>
Analysis summary shows the number of regions processed and the number of objects deleted from the image
