# Supplementary material for: Hairiness: the missing link between pollinators and pollination
Source: PeerJ. 2016 Dec 21;4:e2779. doi: 10.7717/peerj.2779 (PMC5180583; doi:10.7717/peerj.2779)
Supplement: Supplemental Information 5 — Variation in entropy values for multiple photos (n = 5 per region per species) for two different body regions of the same specimen. [file peerj-04-2779-s005.docx]

|  | **Face** | | | | **Thorax dorsal** | | | | |
| --- | --- | --- | --- | --- | --- | --- | --- | --- | --- |
|  | Mean | Mean (*n*=2) | SD | Range | | Mean | Mean (*n*=2) | SD | Range |
| *Bombus terrestris* | 156.3 | 155.9 | 1.0 | 2.3 | | 134.4 | 134.2 | 2.4 | 6.4 |
| *Leioproctus* sp. | 157.2 | 158.9 | 2.0 | 4.8 | | 137.0 | 137.5 | 2.1 | 5.9 |
