# Supplementary figures and images for: Hairiness: the missing link between pollinators and pollination
Source: PeerJ. 2016 Dec 21;4:e2779. doi: 10.7717/peerj.2779 (PMC5180583; doi:10.7717/peerj.2779)

Stigma

Petal

a.

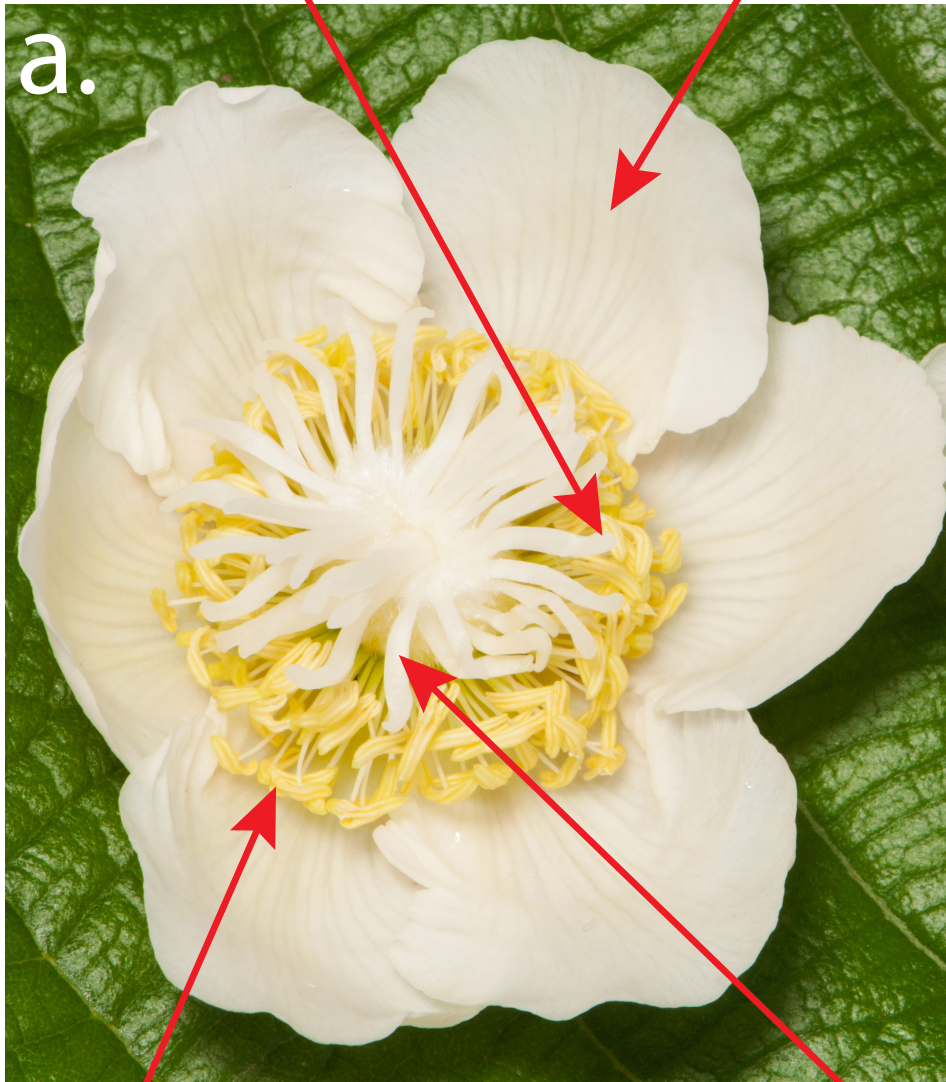

Petal

Anther

b.

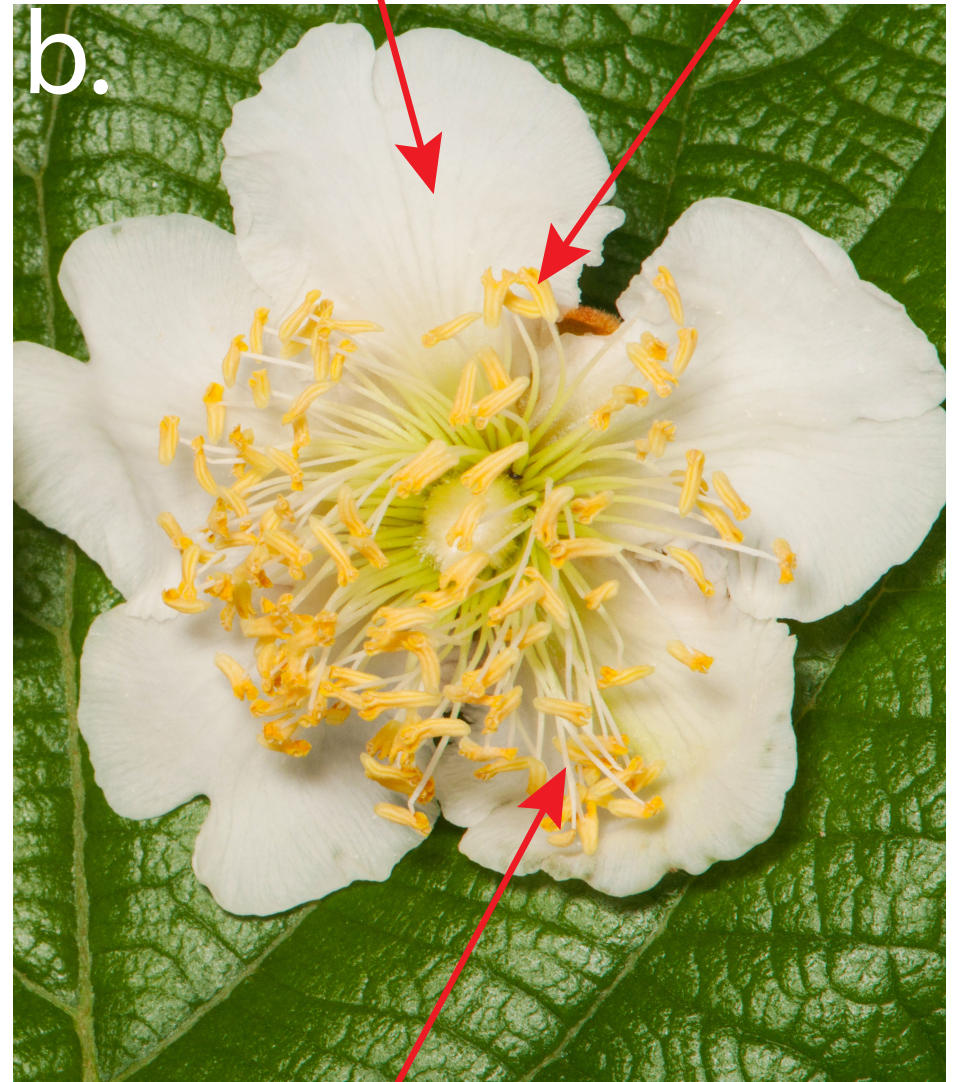

Anther

Style

Filament

Supplement: Figure S3 — Photographs of a female (a) and male (b) kiwifruit Actinidia deliciosa flower. Labels show the key reproductive structures. [file peerj-04-2779-s009.pdf]
